# OUTCOME MEASURES IN MUSCULAR DYSTROPHY REHABILITATION: AN ICF CONTENT COMPARISON APPROACH TO THE MOST COMMONLY USED MD SCALES

**DOI:** 10.2340/jrm.v57.40327

**Published:** 2025-01-31

**Authors:** Mihaela TARANU, Raquel SEBIO-GARCÍA, José C. MILISENDA, Aida ALEJALDRE, Xavier PASTOR, Sara LAXE

**Affiliations:** 1 Physical Medicine and Rehabilitation Department, Hospital Germans Trias I Pujol, Badalona, Barcelona, Spain; 2 WHOFIC Academic Collaborating Center-Universitat de Barcelona, Barcelona, Spain; 3 Muscle Research Unit, Internal Medicine Service, Hospital Clínic de Barcelona (HCB), Universidad de Barcelona and Center for Biomedical Research on Rare Diseases (CIBERER), Barcelona, Spain; 4 Neurology Service, Hospital Clinic, Barcelona, Spain; 5 August Pi i Sunyer Biomedical Research Institute (IDIBAPS) University of Barcelona, Barcelona, Spain; 6 Clinical Informatics, Hospital Clínic – Universitat de Barcelona, Barcelona, Spain; 7 Physical and Rehabilitation Department, Hospital Clinic, ICEMEQ, Barcelona, Spain; 8 Clinical and Experimental Respiratory Immunoallergy (IRCE), Clinic Foundation for Biomedical Research, Barcelona, Spain

**Keywords:** muscular dystrophy, outcome measures, international classification of functioning, functional status

## Abstract

**Introduction:**

Functioning is the reason to be of rehabilitation as it is essential to the lives of people who suffer from a disease. The International Classification of Functioning, Disability and Health (ICF) can help in designing a functioning profile of a patient, identifying needs for rehabilitation plans and measuring the results of an intervention.

**Objective:**

To identify the outcome measurement instruments reported in clinical studies in muscular dystrophies (MDs) and provide an ICF content analysis.

**Method:**

A systematic literature review was conducted until October 2022, using Medline, PubMed, and Scopus databases. Papers assessing outcomes related to functioning in patients with MDs were included.

**Results:**

A total of 116 papers were included and all identified outcome measures were linked to the ICF. Inter-researcher agreement for the linking process was 0.82. The analysed instruments focused mainly on aspects of body functions, followed by activities and participation. General scales were more comprehensive than specific.

**Conclusions:**

The application of ICF in this research enhances the understanding of functioning of patients with MDs and the need to develop more specific but comprehensive scales tailored to the needs of MD patients, and can guide clinicians in a assessing patients through a biopsychosocial perspective.

## LAY ABSTRACT

Muscular dystrophies (MDs) are neuromuscular diseases for which no curative treatment exists to date and that occasion a wide spectrum of functional deficits. Functioning is the reason to be of rehabilitation. International Classification of Functioning (ICF) can help in designing patients’ functioning profile, identifying needs for rehabilitation plans and measure the results. We conducted a systematic literature review identifying a broad variety of outcome measurement instruments reported in clinical studies and provide an ICF content analysis. The instruments focused on body functions, followed by activities and participation. The application of ICF enhances the understanding of functioning of patients with MDs and the need of developing more specific but comprehensive scales tailored to the needs of MDs patients as well as can guide clinical assessment through a biopsychosocial perspective.

Muscular dystrophies (MDs) encompass a diverse spectrum of inherited disorders, hallmarked by a progressive decline in muscular strength and integrity. These conditions are the result of genetic aberrations affecting the coding sequences responsible for producing muscular proteins, both structural and functional in nature. The clinical manifestations of MDs extend beyond mere muscular impairment. Research has identified a constellation of additional symptoms including respiratory failure, cardiac arrhythmias, sensory deficits, hormonal irregularities, or cognitive dysfunction (1).

MDs have variable clinical presentations between patients but also within the same person as time passes. In cases of family transmission, penetrance varies with the debut of symptomatology at different ages and occasioning different grades of disability. Problems in self-care, hygiene, feeding, dressing, or locomotion are frequently reported by patients with MDs, but so are restrictions in social participation such as schooling, working, achieving and maintaining financial independence, family duties, or social activities (2, 3). The result is impaired functional capacity with a negative impact on personal autonomy, activity and participation, and overall quality of life, especially if we consider that MDs also affect children and young persons and that they can experience a change in functioning across their lifespan (4, 5).

To craft effective rehabilitation strategies, a comprehensive assessment of a patient’s functional status is imperative (6–8). Clinicians are tasked with the identification of physical and psychological impairments using assessment tools that are not only valid and reliable but also capable of consistently reflecting changes in function over time or in response to targeted interventions (9, 10).

The International Classification of Functioning, Disability and Health (ICF), established in 2001 (11), aspires to be the universal framework for the assessment and categorization of functioning. Within this model, functioning is depicted as an amalgamation of the interactions between an individual with a health condition and his/her environmental context – comprising both personal and external factors. The ICF delineates 4 principal domains of functioning: body functions, body structures, activities and participation, and environmental factors. The ICF provides a framework to solve the existing gap between the biomedical parameters and a multidimensional approach that considers other constructs of disability and health (8).

Acknowledging this need, the World Health Organization (WHO) has encouraged the use of the ICF and the perspective of functioning for the study of numerous health conditions. Recent initiatives such as development of the ICF core sets (12, 13), which already exist for different conditions like traumatic brain injury, breast cancer, spinal cord injury, and multiple sclerosis, or the WHO Package of Interventions for Rehabilitation, try to provide the use of a broad view of health and the use of outcome measures that capture the full spectrum of functioning (14–18).

The primary objective of this study is to identify how functioning is measured in MDs according to the scientific literature and, second, to use the ICF to categorize the concepts across the different functioning domains of the biopsychosocial model.

## Materials and methods

A protocol for this systematic review was registered in PROSPERO under the registration code CRD42022357257 and was conducted according to the latest PRISMA guidelines. In this systematic review, we aimed to answer the following research question: Which outcome measurements are used in patients with MDs by the researchers in the field? From the identified outcomes the meaningful concept was extracted and linked to the domains of the ICF.

Inclusion criteria included: (a) cohorts of patients diagnosed with any form of MD, with at least 50% of participants over 18 years old; (b) assessment of any aspect of the patients’ functionality (muscle strength, gait evaluation, balance, upper limb function and object manipulation, activities of daily living, aerobic capacity); (c) original research studies (longitudinal, cross-sectional., and controlled trials were all considered); (d) articles published between 2012 and 2022.

The exclusion criteria were conference proceedings and communications, studies involving animals, and studies that focused only on the structure and not function of the patients (i.e., diagnostic imaging tests, electrophysiology, analyses, etc.). Only studies published in English or Spanish were considered for inclusion.

### Search strategy

Before conducting the systematic review, a comprehensive search was conducted in the Cochrane Library Plus and the PROSPERO registry to ensure that no other similar systematic review was done.

The systematic literature review was then conducted using Medline, Scopus, and Cochrane until 31 October 2022. We followed the methodology recommended by ICF Research Branch: (i) literature search to identify the studies that evaluate functioning in patients with MDs, (ii) study selection on the basis of the inclusion criteria, (iii) extraction of relevant concepts (the outcome measures), (iv) identification of meaningful concepts and linkage of those concepts to the ICF (19). Although frequency of analysis was initially intended, outcome measures such as MD-specific scales, assessments related to speech and swallowing, or cognitive function did not meet the 5% threshold. Consequently, we decided to include all identified outcomes regardless of frequency. The search terms “Muscular Dystrophy” AND “Assessment” OR “Evaluation” were combined with a series of terms related to function such as “Gait, Postural Balance”, “Activities of Daily Living”, “Muscle Strength” “Upper extremity Function”. The full search strategy can be found in Table I for each database. A cross-manual search was also conducted among the identified studies and reviewers’ personal records.

**Table I:** Search strategy

Database	Search fields	Search terms
PubMed	Mesh headings Title/Abstract	1. Gait 2. Postural balance 3. Work capacity evaluation
MEDLINE (Ovid)	Mesh headings Abstract	4. Activities of daily living 5. Physical endurance 6. Muscle strength 7. Muscle dystrophy 8. Assessment 9. Evaluation
SCOPUS	Title, Abstract, Keywords	1. Functional capacity 2. Gait 3. Balance 4. Activities of daily living 5. Exercise tolerance 6. Exercise endurance 7. Muscle strength 8. Muscular dystrophy

Two independent investigators (MT and RS) performed the initial search against inclusion and exclusion criteria. After removing duplicates, a selection based on title and/or abstract was conducted. In case of disagreement, this was solved by a third researcher (SL). The Rayyan® online platform (https://www.rayyan.ai/) was used to store, manage, and screen abstracts for inclusion. Data extraction was performed using a spreadsheet (Microsoft Corp^®^, Redmond, WA, USA).

All measurement instruments used in the selected studies were then identified and the number of studies in which these measures appeared was documented.

The concepts contained in the items or tasks addressed in each of the measurement instruments were linked to the ICF categories, following standardized linking rules set out by Cieza et al. (20). This means that each item of a measure should be linked to the most precise ICF category. For example, from the item “In the face of my condition, the way I look is …” the meaningful concept was linked to body image (b1801) from the ICF body function and structure domain. Some items may have not sufficient information to decide, and “nd” (not definable) would be assigned. If the item is not contained in the ICF, the “nc” (not covered) would be defined.

All steps of the systematic review were done by 2 health professionals (MT, physician with specialization in physical medicine and rehabilitation, and RS, physical therapist), under the supervision of SL (physician with specialization in physical medicine and rehabilitation that was trained at the ICF Research Branch, WHO FIC Collaborating Center at the Ludwig Maximilian University in Munich).

The degree of agreement between the 2 professionals was calculated by means of kappa statistics; the values of kappa range from 0 to 1, where 1 means perfect agreement.

## Results

### Search results

**Fig. 1:**
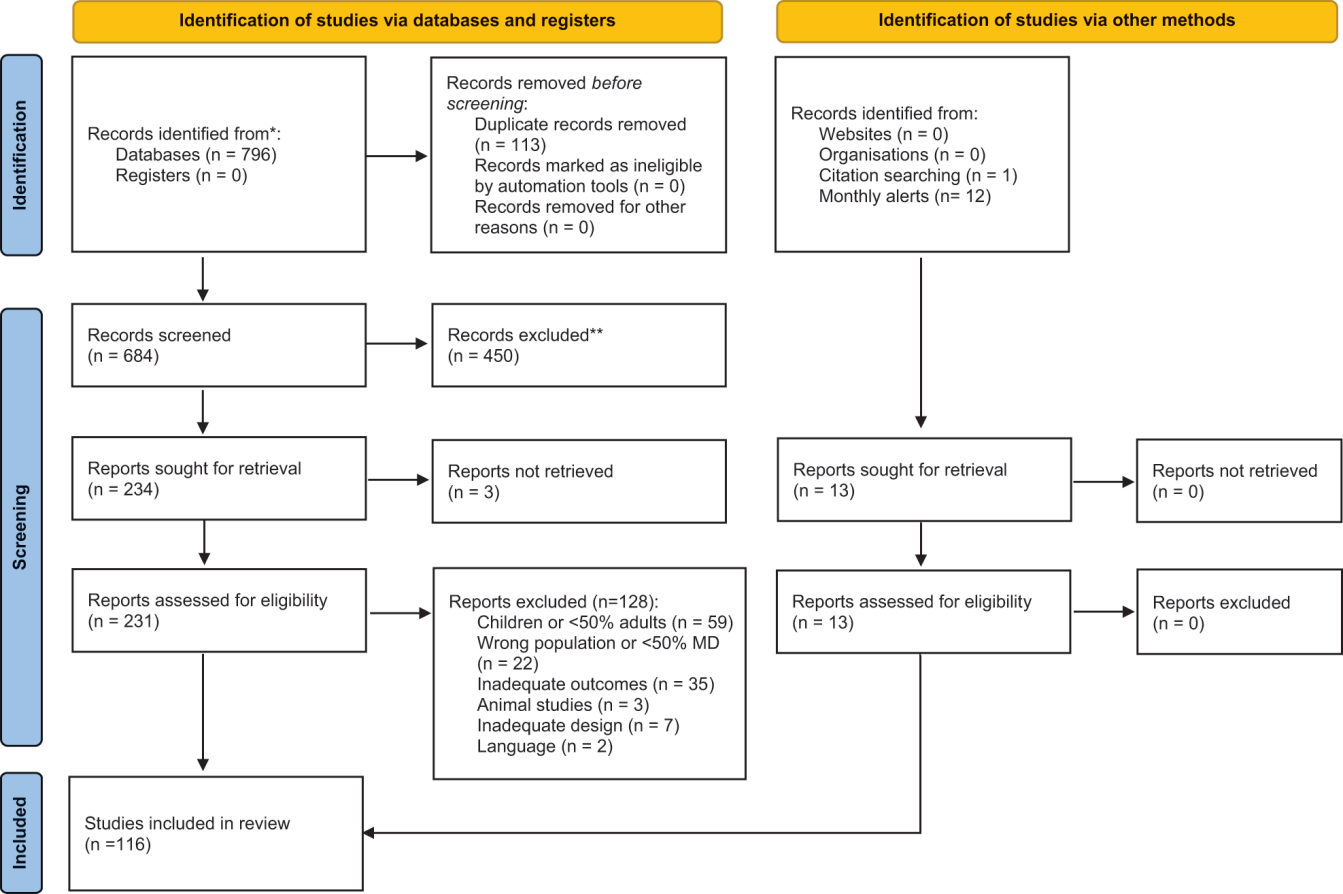
Flow diagram based on PRISMA 2020 for the literature review. From: Page MJ, McKenzie JE, Bossuyt PM, Boutron I, Hoffmann TC, Mulrow CD, et al. The PRISMA 2020 statement: an updated guideline for reporting systematic reviews. BMJ 2021; 372: n71. doi: 10.1136/bmj.n71. For more information, visit: http://www.prisma-statement.org/

A flow diagram of the study selection process is shown in Fig. 1. The initial search in the databases yielded a total of 796 records. No additional record was identified from the cross-manual search. After removing duplicates, screening by title/abstract and eventually by full-text assessment, a total of 116 studies were included in the systematic review. Considering that one of the inclusion criteria was the age of patients (older than 18 years), the great majority of studies with patients with Duchenne muscular dystrophy (DMD) (the most prevalent dystrophy) were discarded.

### Participants, study characteristics, and design

Of 116 articles, the majority focused on population diagnosed with myotonic dystrophy (MyD) (*n* = 43) or facioscapulohumeral dystrophy (FSHD) (*n* = 42) (the 2 most prevalent MDs, after DMD). The remaining papers had patients diagnosed with other types of dystrophies such as limb girdle muscular dystrophy (*n* = 12) or DMD and Becker muscular dystrophy (*n* = 16), or a combination of different phenotypes. Mean age of the participants when reported was 41.4 years and approximately 64% were men. Forty-seven out of 116 studies were of a cross-sectional design, 44 studies were description of a series of cases, 9 nine RCTs and 2 nRCTs, 4 case-control studies, and 1 cohort study as well as 9 systematic reviews.

### Interrater reliability

Initial agreement between reviewers for the first round was 0.63 (moderate agreement). After full-text assessment, agreement reached 0.82 (good agreement). Disagreements were finally solved by a third reviewer (SL).

### Outcomes

In the review, 142 measurement instruments or tests were identified. The instruments vary widely, including both generic tools and those tailored to specific types of muscle dystrophies.

The generic instruments covered a broad spectrum of evaluations that included: tests for evaluation of muscle force, upper extremity and related function, lower extremity and related function, stance, and balance, visual analogue scale for pain and fatigue, respiratory functions, cognitive function such as speech, swallowing, depression, and quality of life.

The research highlighted a range of specific instruments designed to assess the severity and impact of MDs.

The research identified specific severity scales for 2 different clinical entities: FSHD and MyD. Notably, these scales have not been widely used, with none reaching a frequency analysis of 5%.

- Ricci’s clinical severity score, for FSHD; this is a 10-grade scale in which 0 indicates no muscle weakness and 10 indicates wheelchair dependency (21).
- FSHD clinical score, a standardized clinical score that quantifies muscle weakness and includes the evaluation of facial involvement, scapular girdle weakness, upper extremity evaluation, leg involvement and pelvic girdle involvement (21).
- FSHD – COM (composite outcome measure), which includes functional assessment of the legs, shoulders and arms, trunk, hands, and balance/mobility (21).
- DM 1 functional rating scale (DM 1 active) for MyD type 1, which is a 20-item scale representing the domain of activity and participation from the ICF, and it covers a great range of activities of daily living and social activities (22).
- Myotonia Behavior scale, which is a rating scale that assess the stiffness, limitations in activities of daily living, and implication of mental functions in patients with MyD (22).

Specific scales to evaluate the quality of life and activity limitations in patients with neuromuscular diseases were identified and are listed below:

- MDQoL Muscular Dystrophy Quality of Life – a specific measure for quality of life (23).
- Individualized Neuromuscular Quality of Life INQoL (24).
- ACTIVLIM (Activity limitations for patients with upper and/or lower limb impairments) is a measure of activity limitations for patients with neuromuscular diseases, with upper and/or lower limb impairments (25). The scale measures patients’ ability to perform daily activities requiring the use of the upper and/or the lower limbs (Table II).

All identified concepts were translated to ICF language, resulting in a total of 65 ICF second-level categories. One category was linked to a third level: b 4552 (fatigability).

**Table II:** Outcome instruments identified in the review, number of articles in which they were found, and frequency analysis

Outcome	Instrument	*n* (total 142) (%)
Muscle function/sStrength	Manual Muscle Testing Instrumental Muscle testing	30 (25.9) 73 (62,9)
ADL/physical activity/participation	Inclusion Body Myositis Functional Rating Scale Rivermead Mobility Index Assessment of Motor and Process Skills Assessment of Life Habits Adult Myopathy Assessment Tool Time to get up from floor Health Assessment Questionnaire Modified Lawton ADL Time to put on a T-shirt ADL Scale Independent Living Scale Activities of Daily Living Profile University of Michigan Upper Extremity Questionnaire International Physical Activity Questionnaire IPAQ Human Activity Profile Utrecht Scale for Evaluation of Rehabilitation Community Integration Questionnaire Impact on Participation and Autonomy Questionnaire	3 (2.6) 3 (2.6) 2 (1.7) 2 (1.7) 1 (0.9) 1 (0.9) 1 (0.9) 1 (0.9) 1 (0.9) 1 (0.9) 1 (0.9) 1 (0.9) 1 (0.9) 1 (0.9) 1 (0.9) 1 (0.9) 1 (0.9) 1 (0.9)
Quality of life/symptoms	SF36 VAS Individualized Neuromuscular Quality of Life (INQoL) PROMIS57 Sickness Impact Profile Quality of Life in Neurological Disorders (NeuroQLQ) Canadian Occupational Performance Measure Facial-Disability Index Brief Symptom Inventory Nottingham Health Profile Daily Observed Pain Brief Pain Inventory Muscular Dystrophy Quality of Life (MDQoL) Swinyard Severity Classification Scale Swedish version of Canadian Occupational Performance Measure (COPM) Assessment of Life Habits questionnaire LIHE H	12 (10.4) 6 (5.2) 3 (2.6) 3 (2.6) 2 (1.7) 1 (0.9) 1 (0.9) 1 (0.9) 1 (0.9) 1 (0.9) 1 (0.9) 1 (0.9) 1 (0.9) 1 (0.9) 1 (0.9) 1 (0.9)
Mood	Checklist Individual Strength Centre for Epidemiologic Studies Depression Scale (CES-D) Beck Depression Inventory Apathy Evaluation Scale Coping Inventory for Stressful Situation (CISS) Dutch Hospital Anxiety and Depression Scale HADS	6 (5.2) 2 (1.7) 1 (0.9) 1 (0.9) 1 (0.9) 1 (0.9)
Upper limb and hand functioning	Brooke Upper Extremity Scale Dynamometry Nine-hole Peg Test ABILHAND Pegboard Test Upper Extremity Functional Index Box and Block Test Capabilities of Upper Extremity Questionnaire Minnesota Dexterity Test MoviPlate Oxford Shoulder Score Assemble peg units Turning blocks Reachable Workspace Upper Limb Functional Ability Test Jebsen Hand Function Test Performance of the Upper Limb (PUL)	4 (3.4) 4 (3.4) 4 (3.4) 3 (2.6) 3 (2.6) 2 (1.7) 2 (1.7) 2 (1.7) 2 (1.7) 2 (1.7) 1 (0.9) 1 (0.9) 1 (0.9) 2 (1.7) 1 (0.9) 1 (0.9) 1 (0.9)
Global motor function	Motor Function Measure (MFM) Functional Independence Measure	9 (7.7) 2 (1.7)
Lower limb functioning, gait, and balance	Walking Tests (6MWT, 2MWT, 10 m/30 feet WT, Time to climb stairs (4 steps, 8 steps, 14 steps, unspecified) Timed Up and Go (TUG) Step test Sit to stand test (variants: 30”, 5 times) Cycle Test (constant or incremental) Accelerometry/Pedometer Get up from the floor Berg Balance Scale Activities-specific Balance Confidence Scale Lower Extremity Functional Scale LEFS North Star Ambulatory Assessment NSAA Vignos Lower Extremity Function Score GAITRite Functional Ambulation Profile-FAP Locometre Balance platform Stabilimeter Functional Reach Test Standing Balance Test Performance-Oriented Mobility Assessment (Balance & Gait)	69 (59.5) 9 (7.7) 13 (11.2) 14 (12.0) 10 (8.8) 9 (7.7) 9 (7.7) 4 (3.5) 9 (7.8) 4 (3.4) 2 (1.7) 4 (3.4) 3 (2.8) 1 (0.9) 1 (0.9) 1 (0.9) 1 (0.9) 1 (0.9) 1 (0.9) 1 (0.9) 1 (0.9)
Severity	Neuromuscular Impairment Function and Disability Scale (NIFDS) Egen Klassification Gardner-Medwin-Walton scale Modified Rankin Scale	1 (0.9) 1 (0.9) 1 (0.9) 1 (0.9)
Lung function	Spirometry PiMax/PeMax SNIP Pletismography Peak Cough Flow Capnography	15 (13) 4 (3.5) 3 (2.6) 2 (1.7) 2 (1.7) 1 (0.9)
Cardiac function	Echocardiography ECG	5 (4.4) 5 (4.4)
Speech and swallowing	Dysphagia specific quality of life SWAL-QOL HAMASCH-5-points test Regensburg Word Fluency Test Iowa Oral Performance Instrument Communicative participation Item Bank Northwestern Dysphagia Patient Check Sheet Speech-Language Pathology Assessment for Dysphagia Risk	2 (1.7) 1 (0.9) 1 (0.9) 1 (0.9) 1 (0.9) 1 (0.9) 1 (0.9)
Sleep and fatigue	Fatigue Severity Scale Epworth Sleepiness Scale Fatigue and Daytime Sleepiness Scale Würburg Fatigue Inventory Fatigue Severity subscale of the Checklist Individual Strength	5 (4.4) 3 (2.6) 1 (0.9) 1 (0.9) 1 (0.9)
Cognitive function	Stroop Test Mini Mental State Examination Trail Making Test Digit Span Test Vineland Adaptative Behavior Scale HAMASCH 5 points test, Regensburg word fluency, Delis Kaplan Executive Function System, test battery of attentional performance (TAP), TMT-A Wechsler Intelligence scale Cognitive Failure Questionnaire CFQ	2 (1.7) 1 (0.9) 1 (0.9) 1 (0.9) 1 (0.9) 1 (0.9) 1 (0.9) 1 (0.9)
Muscular dystrophy specific instruments	FSHD-COM FSHD-Health Index Ricci’s Clinical Severity Scoring FSHD Clinical Score FSHD – functional composite COA Activity and Social Participation DM1-Active Scale ACTIVLIM FSHD Rasch-Built Overall Disability Scale DMD Functional Ability Self-Assessment Tool Muscular Dystrophy Functional Rating Scale Adult Myopathy Assessment Tool Myotonic Dystrophy Health Index Modified Functional Disability Scale Myotonia Behavior Scale	2 (1.7) 2 (1.7) 2 (1.7) 2 (1.7) 2 (1.7) 2 (1.7) 2 (1.7) 1 (0.9) 1 (0.9) 1 (0.9) 1 (0.9) 1 (0.9) 1 (0.9) 1 (0.9)
Other	ALCOS16 Toronto Alexithymia Scale (TAS-20) General Self-Efficacy Scale Scale of Assessment and Rating of Ataxia (SARA) Karnofsky index	1 (0.9) 1 (0.9) 1 (0.9) 1 (0.9) 1 (0.9)

We analysed meaningful concepts from MDs-specific outcome measures (FSHD-COM, DM1-Activ, INQoL, Ricci’s score, FSHD clinical scale) and translated them to ICF language; those items that were mentioned once or twice were marked with x, and those that were referred to approximately 3 or more times were marked xxx.

For non MDs-specific instruments (general outcome measurements) we did the same. For example, “dysphagia” was found in 3 scales: the dysphagia-specific quality of life scale (SWAL-QOL), Northwestern Dysphagia Patient Check Scale, Speech-Language Pathology Assessment for Dysphagia Risk; none of these scales is MDs specific, so we rated it xxx in the first column (General and unspecific neuromuscular scale). In the specific MD scale, in the INQoL there are 3 items regarding dysphagia, so we attributed this xxx.

Most of the concepts referred to activity and participation (35 categories), followed by 26 in the domain of body functions and 4 in environmental factors as seen in Tables III–VI All the concepts were able to be linked to ICF.

**Table III:** Content comparison of outcome measures based on the ICF linking for body structures, identifying a number of 8 ICF categories (*n*=8)

ICF code	Explanation	MDs specific scales	General scales
s 2308	Structures around eye, other specified (lid)	x	x
s 3204	Structure of lips	x	x
s 3208	Structure of mouth, other specified (cheeks)	x	x
s 7104	Muscle of head and neck region	x	–
s 7201	Joints of shoulder region	x	x
s 730	Structure of upper extremity	x	–
s 750	Structure of lower extremity	x	–
s 760	Structure of trunk	x	–

x: 1 or 2 items included, xxx: 3 or more items included.

No item was identified as being mentioned 3 or more times.

**Table IV:** Content comparison of outcome measures based on the ICF linking for body functions, identifying a number of 26 ICF categories (*n*=26)

ICF code	Explication	General and unspecific neuromuscular scales	FSHD-COM	DM 1- Activ	INQoL	Ricci’s score	FSHD clinical scale
b114	Orientation functions	x					
b 1801	Body image				xxx		
b 130	Energy and drive functions	xxx			xxx		
b 134	Sleep functions	x					
b140	Attention functions	x					
b144	Memory functions	x					
b 152	Emotional functions	xxx			xxx		
b 164	Higher-level cognitive functions	xxx					
b 210	Seeing functions	x			xxx		x
b215	Functions of structures adjoining the eye				x		
b230	Hearing functions				x		
b235	Vestibular functions	xxx	x	x			
b 280	Sensation of pain	xxx			xxx		
b 398	Other specified voice and speech functions	x					
b440	Fine hand use	xxx		x			
b 455	Exercise tolerance functions (b4552 fatigability)	xxx		x	xxx		
b 510	Ingestion functions				xxx		
b 515	Digestive functions	x					
b 525	Defecation functions	x					
b 620	Urination functions	x					
b 640	Sexual functions	x					
b710	Mobility of joints	xxx	xxx			x	
b 730	Muscle power functions	xxx	xxx		xxx	xxx	xxx
b 740	Muscle endurance function	x			xxx	x	
b 76o	Control of voluntary movement function	x			xxx		
b 770	Gait pattern function	x	x	xxx		x	x

x:1 or 2 items included, xxx: 3 or more items included.

**Table V:** Content comparison of outcome measures based on the ICF linking for activity and participation, identifying a number of 35 ICF categories (*n*=35)

ICF code	Explication	General scales	DM 1- Activ	INQoL	Ricci’s score	FSHD-COM	ACTIVLIM
d 110	Watching	x					
d 115	Listening	x					
d160	Focusing attention	x					
d 175	Solving problems	x					
d 177	Making decisions	x					
d 230	Carrying out daily routine	x	x				
d 240	Handling stress and other psychological demands	xxx		x			
d 330	Speaking	x					
d 350	Conversation	x					
d 410	Changing basic body position	x	x		x	x	xxx
d 415	Maintaining body position	x	x				
d 420	Transferring oneself	xxx					xxx
d 430	Lifting and carrying objects	x					x
d 435	Moving objects with lower extremities	x					
d 440	Fine hand use	x	x				x
d 445	Hand and arm use	x	x		x		x
d 449	Carrying, moving, and handling objects, other specified and unspecified	x	x				
d 450	Walking	x	x		x	x	x
d 451	Going up and down stairs					x	x
d 455	Moving around	x	x		x	x	x
d 470	Using transportation	x	x				x
d 510	Washing oneself	x	X	x			
d 520	Caring for body parts	x	x	x			xxx
d 540	Dressing						x
d 598	Other specified self-care	x	x	x			x
d 620	Acquisition of goods and services	x					
d 630	Preparing meals	x	x				
d 640	Doing housework	x	x	x			
d 710	Basic interpersonal interaction	xxx					
d 770	Intimate relationships	x		x			
d 850	Remunerative employment	x	x	x			
d839	Education	xxx					
d 920	Recreation and leisure	x	x	x			x
d 940	Human rights	x					
d 950	Political life and citizenship	x					

x: 1 or 2 items included, xxx: 3 or more items included.

**Table VI:** Content comparison of outcome measures based on the ICF linking for environmental factors, identifying 4 ICF categories (*n*=4)

ICF code	Explication	General scales	Specific MDs scales
e 115	Products and technology for personal use in daily living	x	
e 120	Products and technology for personal indoor and outdoor mobility and transportation	x	
e 125	Products and technology for communication	x	
e 320	Friends	x	xxx

x: 1 or 2 items included, xxx: 3 or more items included.

In the specific MDs scales, only INQoL refers to social relationships category (3 items in this questionnaire are dedicated to friends and friendships).

## DISCUSSION

This systematic review of the literature has revealed a wide variety of measurement instruments used in the study of MDs, and these findings are consistent with other similar studies with other health conditions (16–18, 26). Both the generic and specific outcome measures identified covered a wide spectrum of problems in functioning, showing that even if dystrophies are originally a muscle disease, the consequences, impairments, and limitations on a person’s life cover a wide spectrum of functioning, not limited to the muscle affectation.

In 2013, Bos et al. published an ICF core set for neuromuscular diseases on the basis of an analysis of 3 specific scales for neuromuscular conditions (Amyotrophic Lateral Sclerosis Assessment Questionnaire, Myasthenia Gravis Quality of Life 60, and Individualized Neuromuscular Quality of Life Questionnaire), extracting the meaningful concepts that were further translated to ICF language (27); in comparison with that study, we looked for scales and instruments used in the research literature, both specific and general, further identifying meaningful concepts in those outcome measures and linking them to the ICF, allowing for ICF-based mapping (13).

Our decision not to include “ICF” in the initial search terms was based on the specific focus of our study on other aspects of MDs measurement scales that may not directly involve ICF.

As expected, the most valued categories are muscle strength, those related to gait, energy and impulses (fatigability), use of the upper extremity, pain sensation and fatigue, and respiratory involvement (regarding the first domain of the ICF – (b) body functions) (Table IV), and the aspects on activity and participation (d) (Table V) like mobility, moving around and self-care, the targeted areas for rehabilitation interventions.

One of the principles of rehabilitation interventions is to overcome impaired body function, activity limitations, and participation restriction, so, as expected, majority of identified categories belong to those 2 domains: (b) body functions and (d) activity and participation.

Regarding the domain of body structures, we noticed a significant omission in ICF categories. This is a striking oversight, given the fact that people who suffer from MDs often experience pronounced changes in their body structure such as muscle atrophy, limited range of motion, even changes in spinal alignment. The scarceness of categories focused on body structures in this review could be attributed to the predominant focus on functional outcomes that could have overshadowed the direct assessment of structural changes. This gap highlights once again the need for a more holistic approach in MDs research and maybe clinical practice.

Our search identified assessment instruments of speech and swallowing function in MDs patients, but with a frequency of analysis of less than 5%, and this is less than expected, especially if we consider that some of these features are characteristic of some entities, for example, in oculopharyngeal dystrophy (DOP): in the late stages almost all patients have dysphagia and in 32% of cases this is the presenting symptom (28).

In MD type 1, 25–80% of patients present with dysphagia, in various grades of severity, depending on the duration and severity of the disease, and it affects the 3 phases of the deglutition. In FSHD, swallowing difficulties are present in 25% of patients (29).

In DMD, the swallowing problems increase as the disease progress, making tube feeding necessary in approximately 22% of patients at a median age of 19 years (30).

Speech and communication problems are secondary to weakness of facial as well as bulbar muscles, and respiratory failure, with a negative impact on the ability to form facial expressions, thus interfering with non-verbal communication; but there are also components of dysphonia and dysarthria, documented in all types of MyD, in FSHD, in DOP, and in Emery Dreifuss (27–31). In DMD, speech and communication problems depend on the severity of the disease, ranging from no problems in the early stages to absence of speech in late stages (32).

A possible explanation for why we found such a small incidence of instruments that assess speech and swallowing may be the search strategy itself: we excluded articles referring to diagnostic techniques. Conceptual analyses like the one presented in this review are valuable for highlighting that some aspects, which may be critical for patients and influence their overall functioning, are often underrepresented in the published literature. For example, while gross motor function, fine motor function, fatigue, and pain are commonly explored, communication and eating disorders may receive less attention despite their potential impact on patients’ lives.

Cognitive deficits are often overlooked as outcome tools for MDs patients, even though this aspect is crucial for proper functioning. From the revised articles the frequency of analysis was less than 1% in tests that evaluate cognitive functions – memory, attention, and verbal fluency – but studies have shown that people affected by DMD are at risk of developing some kind of neurocognitive deficit. This is also true for patients diagnosed with MyD, DOP, and FSHD (33–37).

The reproductive aspects are also part of the functioning of a person, but the search with key words regarding functioning did not identify outcome measures that address this domain although, dysfunctions of the reproductive cells in men and women with MyD have been described (38, 39). Nevertheless, this aspect can be covered by categories from ICF domains, permitting the completion of the functioning profile of the person.

Aspects of contextual environmental factors (e), have a scarce representation (Table VI) despite being important factors in the patient’s functioning. The identification of contextual factors, even though they cannot be qualified as outcome measures, is important as they can act as barriers or facilitators and affect the overall outcome. The work by Bos et al. also highlights the importance of integrating categories from the domain of activity and participation as well as environmental factors, which are often underrepresented in traditional HRQoL instruments, in order to achieve a more holistic understanding of patient challenges. This perspective aligns with our current review, which emphasizes that addressing the full spectrum of patient functioning, and understanding functioning in the ICF whole model, can enhance the effectiveness of interventions and health policies tailored to neuromuscular conditions

One explanation for the lack of reports on environmental factors could be the exclusion of studies on the use of technology in people with MDs, which could have identified facilitating environmental factors (40, 41).

A key distinction from the ICF checklist developed by Bos et al. lies in the broader range of the categories identified in this review, which highlight aspects warranting attention in the assessments of MDs. Notable examples are body image (b1114) – especially in MDs affecting face musculature; high cognitive level functions (b164) – as mentioned above, an unneglectable percentage of patients with different MDs present neurocognitive deficits; functions of structures adjoining the eye (b215), such as eyelid ptosis; mobility of joints (b710), as most of MDs develop limitations due to tendon retractions. Additionally, categories such as carrying out daily routine (d230) and carrying, moving, and handling objects (d449) address essential functional limitations often observed in these patients.

The application of the ICF and the linkage procedure allow for the studying of the heterogeneity of the measurement instruments used in the studies on MDs, and thus makes possible the development of a checklist of items to look for in evaluation of persons suffering from MDs. It helps to collect detailed information on aspects to be studied and prevents gaps as well as redundancy of data in the instruments used. The ICF offers a comprehensive contextual framework suitable for use both in daily practice and in research; it focuses on “what” needs to be measured, leaving the choice of “how” to measure to the professional. The development of a basic core of ICF categories will help in designing a complete measurement instrument that could cover aspects which are currently underrepresented in measurement instruments, such as cognitive aspects, items related to speech and swallowing, sexual and reproductive function, etc.

In the field of rehabilitation, our approach deliberately avoids a singular focus on specific diseases. Instead, we choose to address the collective group of dystrophies, recognizing them as similar conditions that share common characteristics and challenges. This decision was made to highlight the relevance of these disorders in rehabilitation practice, as many aspects may not be as well represented in the existing literature. While we acknowledge that certain issues within specific dystrophies may be more extensively studied, our aim is to explore the shared implications for rehabilitation across these conditions. By doing so, we hope to provide a comprehensive perspective that is beneficial for practitioners working with patients experiencing MDs.

There are some limitations to our study: only English and Spanish manuscripts were selected; the search was limited to studies from the last 10 years; the infantile population was omitted, thus excluding patients affected by the most prevalent dystrophy (DMD), articles regarding equipment were also excluded, thus probably omitting important facilitator environmental factors.

In conclusion, this study’s utilization of the ICF in assessing functioning in adults with MDs has been revelatory. It was observed that general scales, which address a broader variety of domains, are more capable of providing an integrative assessment. These insights are invaluable for clinical practitioners, enabling them to make more informed decisions when selecting the most appropriate measurement instruments for their patients. Furthermore, the findings serve as a foundational resource for researchers, guiding the development of more specific and comprehensive scales tailored to MDs. The application of the ICF framework in this research not only enhances the understanding of functional health in MDs but also bridges the gap between research and clinical practice, ensuring that assessment tools are both relevant and effective in addressing the multifaceted nature of these conditions.

This work could be a first step in the development of the basic core of what to measure when patients with MDs come for consultation, and how to integrate the vision of the professionals and the patient’s perspective.

## References

[1] Mercuri E, Muntoni F. Muscular dystrophies. Lancet 2013; 381: 845–860. 10.1016/S0140-6736(12)61897-2 23465426

[2] Lindsay S, Cagliostro E, McAdam L. Meaningful occupations of young adults with muscular dystrophy and other neuromuscular disorders. Can J Occup Ther 2019; 86: 277–288. 10.1177/0008417419832466 31096763

[3] Minis MA, Satink T, Kinébanian A, Engels JA, Heerkens YF, van Engelen BG, et al. How persons with a neuromuscular disease perceive employment participation: a qualitative study. J Occup Rehabil 2014; 24: 52–67. 10.1007/s10926-013-9447-8 23645359

[4] Llamosas-Falcón L, Sánchez-Díaz G, Gallego E, Villaverde-Hueso A, Arias-Merino G, Posada de la Paz M, et al. A population-based study of mortality due to muscular dystrophies across a 36-year period in Spain. Sci Rep 2022: 8; 12: 3750. 10.1038/s41598-022-07814-z 35260676 PMC8904487

[5] Natera-de Benito D, Foley AR, Domínguez-González C, Ortez C, Jain M, Mebrahtu A, et al. Association of initial maximal motor ability with long-term functional outcome in patients with COL6-related dystrophies. Neurology 2021; 96: e1413–1424. 10.1212/WNL.0000000000011499 33441455 PMC8055317

[6] Grisbrook TL, Stearne SM, Reid SL, Wood FM, Rea SM, Elliott CM. Demonstration of the use of the ICF framework in detailing complex functional deficits after major burn. Burns J Int Soc Burn Inj 2012; 38: 32–43. 10.1016/j.burns.2011.04.001 22079536

[7] Cieza A, Boldt C, Ballert CS, Eriks-Hoogland I, Bickenbach JE, Stucki G. Setting up a cohort study on functioning: deciding what to measure. Am J Phys Med Rehabil Assoc Acad Physiatr 2011; 90: S17–28. 10.1097/PHM.0b013e318230fddb 21975673

[8] Crawford L, Maxwell J, Colquhoun H, Kingsnorth S, Fehlings D, Zarshenas S, et al. Facilitators and barriers to patient-centred goal-setting in rehabilitation: A scoping review. Clin Rehabil 2022; 36: 1694–1704. 10.1177/02692155221121006 36017567 PMC9574028

[9] Cieza A, Kwamie A, Magaqa Q, Paichadze N, Sabariego C, Blanchet K, et al. Framing rehabilitation through health policy and systems research: priorities for strengthening rehabilitation. Health Res Policy Syst 2022; 20: 101. 10.1186/s12961-022-00903-5 36127696 PMC9487068

[10] Alford VM, Ewen S, Webb GR, McGinley J, Brookes A, Remedios LJ. The use of the International Classification of Functioning, Disability and Health to understand the health and functioning experiences of people with chronic conditions from the person perspective: a systematic review. Disabil Rehabil 2015; 37: 655–666. 10.3109/09638288.2014.935875 24986707

[11] Leonardi M, Lee H, Kostanjsek N, Fornari A, Raggi A, Martinuzzi A, et al. 20 Years of ICF–International Classification Of Functioning, Disability and Health: uses and applications around the world. Int J Environ Res Public Health 2022; 19: 11321. 10.3390/ijerph191811321 36141593 PMC9517056

[12] Kiekens C, Meyer T, Gimigliano F, Baffone C, Gutenbrunner CM; UEMS PRM ICF Workshop moderators and rapporteurs. European initiative for the application of the International Classification of Service Organization in Health-related Rehabilitation (ICSO-R). Eur J Phys Rehabil Med 2017; 53: 308–318. 10.23736/S1973-9087.16.04437-3 28488426

[13] Karlsson E, Gustafsson J. Validation of the International Classification of Functioning, Disability and Health (ICF) core sets from 2001 to 2019 – a scoping review. Disabil Rehabil 2022; 44: 3736–3748. 10.1080/09638288.2021.1878562 33535017

[14] Laxe S, Cieza A, Castaño-Monsalve B. Rehabilitation of traumatic brain injury in the light of the ICF. NeuroRehabilitation 2015; 36: 37–43. 10.3233/NRE-141189 25547765

[15] Package of interventions for rehabilitation. Module 3. Neurological conditions. Geneva: World Health Organization; 2023 (Package of interventions for rehabilitation). Licence: CC BY-NC-SA 3.0 IGO. Available from: http://apps.who.int/iris.

[16] Brach M, Cieza A, Stucki G, Füssl M, Cole A, Ellerin B, et al. ICF Core sSets for breast cancer. J Rehabil Med Off J UEMS Eur Board Phys Rehabil Med 2004; Suppl 44: 121–127. 10.1080/16501960410016811 15370759

[17] Cieza A, Kirchberger I, Biering-Sørensen F, Baumberger M, Charlifue S, Post MW, et al. ICF Core sets for individuals with spinal cord injury in the long-term context. Spinal Cord 2010; 48: 305–312. 10.1038/sc.2009.183 20065984

[18] Laxe S, Zasler N, Selb M, Tate R, Tormos JM, Bernabeu M. Development of the International Classification of Functioning, Disability and Health core sets for traumatic brain injury: an international consensus process. Brain Inj 2013; 27: 379–387. 10.3109/02699052.2012.750757 23472615

[19] Selb M, Gimigliano F, Prodinger B, Stucki G, Pestelli G, Iocco M, et al. Toward an International Classification of Functioning, Disability and Health clinical data collection tool: the Italian experience of developing simple, intuitive descriptions of the Rehabilitation Set categories. Eur J Phys Rehabil Med 2017; 53: 290–298. 10.23736/S1973-9087.16.04250-7 27858402

[20] Cieza A, Geyh S, Chatterji S, Kostanjsek N, Ustün B, Stucki G. ICF linking rules: an update based on lessons learned. J Rehabil Med Off J UEMS Eur Board Phys Rehabil Med 2005; 37: 212–218. 10.1080/16501970510040263 16024476

[21] Ghasemi M, Emerson CP, Hayward LJ. Outcome measures in facioscapulohumeral muscular dystrophy clinical trials. Cells 2022; 11: 687. 10.3390/cells11040687 35203336 PMC8870318

[22] Hermans MCE, Faber CG, De Baets MH, de Die-Smulders CEM, Merkies ISJ. Rasch-built myotonic dystrophy type 1 activity and participation scale (DM1-Activ). Neuromuscul Disord NMD 2010; 20: 310–318. 10.1016/j.nmd.2010.03.010 20363134

[23] Endo M, Odaira, K, Ono R, Kurauchi G, Koseki A, Goto M, et al. Health-related quality of life and its correlates in Japanese patients with myotonic dystrophy type 1. Neuropsychiatr Dis Treat 2019; 15: 219–226. 10.2147/NDT.S187607 30679907 PMC6338121

[24] Seesing FM, van Vught LE, Rose MR, Drost G, van Engelen BGM, van der Wilt GJ. The individualized neuromuscular quality of life questionnaire: cultural translation and psychometric validation for the Dutch population. Muscle Nerve 2015; 51: 496–500. 10.1002/mus.24337 25042897

[25] Fossmo HL, Ørstavik K, Frich JC, Robinson HS. Translation, reliability, and validity of the Norwegian version of the ABILHAND-NMD and the ACTIVLIM for Myotonic Dystrophy type 1. Disabil Rehabil 2024; 46: 2699–2707. 10.1080/09638288.2023.2231848 37438996

[26] Laxe S, Tschiesner U, Zasler N, López-Blazquez R, Tormos JM, Bernabeu M. What domains of the International Classification of Functioning, Disability and Health are covered by the most commonly used measurement instruments in traumatic brain injury research? Clin Neurol Neurosurg 2012; 114: 645–650. 10.1016/j.clineuro.2011.12.038 22245447

[27] Bos I, Stallinga HA, Middel B, Kuks JB, Wynia K. Validation of the ICF core set for neuromuscular diseases. Eur J Phys Rehabil Med 2013; 49: 179–187. 23172408

[28] Kroon RHMJM, Horlings CGC, de Swart BJM, van Engelen BGM, Kalf JG. Swallowing, chewing and speaking: frequently impaired in oculopharyngeal muscular dystrophy. J Neuromuscul Dis 2020; 7: 483–494 10.3233/JND-200511 32804098 PMC7592669

[29] Mul K, Berggren KN, Sills MY, McCalley A, van Engelen BGM, Johnson NE, et al. Effects of weakness of orofacial muscles on swallowing and communication in FSHD. Neurology 2019; 92: e957–63. 10.1212/WNL.0000000000007013 30804066 PMC6404471

[30] Yamada Y, Kawakami M, Wada A, Fukui S, Haruyama K, Otsuka T, et al. Long-term follow-up of dysphagia in adult patients with Duchenne muscular dystrophy. Eur J Paediatr Neurol 2018, 786–790. 10.1016/j.ejpn.2018.06.004

[31] Heller SA, Shih R, Kalra R, Kang PB. Emery-Dreifuss muscular dystrophy. Muscle Nerve 2020; 61: 436–448. 10.1002/mus.26782 31840275 PMC7154529

[32] Hijikata N, Kawakami M, Wada A, Ikezawa M, Kaji K, Chiba Y, et al. Assessment of dysarthria with Frenchay dysarthria assessment (FDA-2) in patients with Duchenne muscular dystrophy. Disabil Rehabil 2022; 44: 1443–1450. 10.1080/09638288.2020.1800108 32772581

[33] Koeks Z, Hellebrekers DMJ, van de Velde NM, Alleman I, Spitali P, van Duyvenvoorde HA, et al. The neurocognitive profile of adults with Becker muscular dystrophy in the Netherlands. J Neuromuscul Dis 2022; 9: 543–553. 10.3233/JND-210770 35723110 PMC9398065

[34] Labayru G, Camino B, Jimenez-Marin A, Garmendia J, Villanua J, Zulaica M, et al. White matter integrity changes and neurocognitive functioning in adult-late onset DM1: a follow-up DTI study. Sci Rep 2022; 12: 3988. 10.1038/s41598-022-07820-1 35256728 PMC8901711

[35] Preethish-Kumar V, Shah A, Polavarapu K, Kumar M, Safai A, Vengalil S, et al. Disrupted structural connectome and neurocognitive functions in Duchenne muscular dystrophy: classifying and subtyping based on Dp140 dystrophin isoform. J Neurol 2022; 269: 2113–2125. 10.1007/s00415-021-10789-y 34505932

[36] Koscik TR, van der Plas E, Long JD, Cross S, Gutmann L, Cumming SA, et al. Longitudinal changes in white matter as measured with diffusion tensor imaging in adult-onset myotonic dystrophy type 1. Neuromuscul Disord NMD 2023; 33: 660–669. 10.1016/j.nmd.2023.05.010 37419717 PMC10529200

[37] O’Dowd DN, Bostock EL, Smith D, Morse CI, Orme P, Payton CJ. The effects of 12 weeks’ resistance training on psychological parameters and quality of life in adults with Facioscapulohumeral, Becker, and Limb-girdle dystrophies. Disabil Rehabil 2022; 44: 5950–5956. 10.1080/09638288.2021.1955306 34340613

[38] Hortas ML, Castilla JA, Gil MT, Molina J, Garrido ML, Morell M, et al. Decreased sperm function of patients with myotonic muscular dystrophy. Hum Reprod Oxf Engl 2000; 15: 445–448. 10.1093/humrep/15.2.445 10655320

[39] Parmova O, Vlckova E, Hulova M, Mensova L, Crha I, Stradalova P, et al. Anti-Müllerian hormone as an ovarian reserve marker in women with the most frequent muscular dystrophies. Medicine (Baltimore) 2020; 99: e20523. 10.1097/MD.0000000000020523 32502004 PMC7306369

[40] Schofield C, Evans K, Young H, Paguinto SG, Carroll K, Townsend E, et al. The development of a consensus statement for the prescription of powered wheelchair standing devices in Duchenne muscular dystrophy. Disabil Rehabil 2022; 44: 1889–1897. 10.1080/09638288.2020.1810786 32878485

[41] Lobo-Prat J, Enkaoua A, Rodríguez-Fernández A, Sharifrazi N, Medina-Cantillo J, Font-Llagunes JM, et al. Evaluation of an exercise-enabling control interface for powered wheelchair users: a feasibility study with Duchenne muscular dystrophy. J Neuroengineering Rehabil 2020; 17: 142. 10.1186/s12984-020-00760-9 PMC7592377 33115472

